# Insulin-Like Peptide and FoxO Mediate the Trehalose Catabolism Enhancement during the Diapause Termination Period in the Chinese Oak Silkworm (*Antheraea pernyi*)

**DOI:** 10.3390/insects12090784

**Published:** 2021-09-01

**Authors:** Ya-Na Li, Xiao-Bing Ren, Zhi-Chao Liu, Bo Ye, Zhen-Jun Zhao, Qi Fan, Yu-Bo Liu, Jia-Ning Zhang, Wen-Li Li

**Affiliations:** 1 School of Bioengineering, Dalian University of Technology, Dalian 116024, China; liyana90@mail.dlut.edu.cn; 2 School of Life and Pharmaceutical Sciences, Dalian University of Technology, Panjin 124211, China; rxb@mail.dlut.edu.cn (X.-B.R.); zhichaoliu@mail.dlut.edu.cn (Z.-C.L.); liuyubo@dlut.edu.cn (Y.-B.L.); jnzhang@dlut.edu.cn (J.-N.Z.); 3 Liaoning Ocean and Fisheries Science Research Institute, Liaoning Academy of Agricultural Sciences, Dalian 116023, China; keyasyie@hotmail.com (B.Y.); zhenzhen1029@126.com (Z.-J.Z.); qifan10001@163.com (Q.F.)

**Keywords:** *Antheraea pernyi*, RNA interference, overexpression, expression profile, trehalose metabolism

## Abstract

**Simple Summary:**

In insects, the insulin/insulin-like growth factor signalling (IIS) pathway regulates the carbohydrate and lipid metabolisms, and plays important roles in diapause regulation. Trehalose accumulates in many diapausing insects, as it is a major carbohydrate reserve and a stress protectant. Because of metabolism depression, the trehalose concentration is maintained at relatively high levels over the diapause phase. In the present study, bovine insulin injection triggered diapause termination and synchronous eclosion in *Antheraea pernyi* pupae. Moreover, treatment with bovine insulin elevated the trehalose catabolism in diapausing pupae. As a homologue of vertebrate insulin, insulin-like peptide (*ApILP*) enhances the trehalose catabolism during the diapause termination process. The transcription factor forkhead box O (*ApFoxO*)—the downstream target of the IIS pathway—exhibited a contrasting effect on the trehalose catabolism to that of *ApILP*. These results suggest that *ApILP* and *ApFoxO* are involved in the regulation of trehalose catabolism during diapause termination in *A. pernyi* pupae.

**Abstract:**

In insects, trehalose accumulation is associated with the insulin/insulin-like growth factor signalling (IIS) pathway. However, whether insulin-like peptide is involved in the regulation of the trehalose metabolism during diapause termination remains largely unknown. This study assessed whether insulin-like peptide (*ApILP*) enhances the trehalose catabolism in the pupae of *Antheraea* *pernyi* during their diapause termination process. Injection of 10 μg of bovine insulin triggered diapause termination and synchronous adult eclosion in diapausing pupae. Moreover, treatment with bovine insulin increased the expression of trehalase 1A (*ApTre-1A*) and trehalase 2 (*ApTre-2*), as well as the activity of soluble and membrane-bound trehalase, resulting in a decline in trehalose levels in the haemolymph. Silencing *ApILP* via RNA interference significantly suppressed the expression of *ApTre-1A* and *ApTre-2*, thus leading to an increase in the trehalose concentration during diapause termination. However, neither injection with bovine insulin nor *ApILP* knockdown directly affected trehalase 1B (*ApTre-1B*) expression. Moreover, overexpression of the transcription factor forkhead box O (*ApFoxO*) induced an increase in trehalose levels during diapause termination; however, depletion of *ApFoxO* accelerated the breakdown of trehalose in diapausing pupae by increasing the expression of *ApTre-1A* and *ApTre-2*. The results of this study help to understand the contributions of *ApILP* and *ApFoxO* to the trehalose metabolism during diapause termination.

## 1. Introduction

To ensure their survival, insects have developed a range of specific abilities to cope with extreme environments. In response to increasing environmental adversity, many insects switch their direct developmental program to diapause [1]. Diapause is a physiological state of developmental arrest, characterized by a depression of the metabolism, which allows individuals to survive under severe conditions of environmental stress [2,3,4]. The management and utilisation of stored energy play important roles in diapausing insects. Energetic reserves accumulate in diapause-destined individuals during the pre-diapause phase [5,6]. To enhance stress resistance, the main energy reserves (which include trehalose, glycogen, and lipids) are consumed in an economical manner because of metabolic depression throughout the diapause stage. In this way, insects can retain sufficient energy reserves to support themselves over an extended diapause period. These reserves have a profound effect on their development following diapause termination [7]. A recent study showed that metabolism enhances in response to diapause transition, suggesting that the change in metabolism may affect the progress of diapause [8].

The Chinese oak silkworm, *Antheraea pernyi*, is a traditional insect resource in China, which is highly valued for its silk as well as its rich nutritional properties [9]. When *A. pernyi* larvae perceive unfavourable environmental signals, such as short daylengths or low temperatures, they are programmed to enter a stage of facultative diapause during their pupal development [10]. As trehalose is a major circulating sugar, and also serves as an agent protecting individuals against environmental stress, its concentration is closely related to the occurrence, maintenance, and termination of diapause in many insect species [11,12,13,14,15]. Previous studies have reported that trehalose accumulates in diapause-destined *A. pernyi* pupae, and relatively high trehalose levels are maintained throughout the diapause phase; however, these levels decrease significantly after diapause termination, suggesting close regulation of the level of trehalose from the onset to the termination of diapause [16,17].

Insulin-like peptide (ILP)—a homologue of insulin—is a crucial controller of the consumption of carbohydrate reserves in insects. ILP has been reported to be involved in regulating haemolymph trehalose levels in various insects. Injection of bombyxin—the first insect ILP that was discovered—decreases the trehalose concentration by elevating trehalase activity in neck-ligated larvae of *Bombyx mori* [18]. Ablation of median neurosecretory cells that produce ILP in the insect brain causes elevated trehalose and glucose levels in the haemolymph of *Drosophila melanogaster* [19]. Similarly, knockdown of insulin-receptor genes significantly increases haemolymph trehalose levels and decreases expression of trehalase in *Maruca vitrata* [20]. The transcription factor forkhead box O (FoxO) is a downstream target, negatively regulated by the insulin/insulin-like growth factor signalling (IIS) pathway, and involved in the regulation of diverse cellular functions, such as differentiation, metabolism, proliferation, and survival [21]. In *Caenorhabditis elegans*, FoxO increases trehalose synthesis by upregulating trehalose synthase expression, which promotes starvation resistance [22]. This evidence suggests that insulin signalling might contribute to the regulation of trehalose levels. Furthermore, a previous study reported that injection of bovine insulin successfully induces diapause termination in the pupae of *Pieris brassicae* [23]. Thus, it was hypothesized that the IIS pathway participates in the regulation of the trehalose metabolism during diapause termination in *A. pernyi* pupae.

In the present study, the genes encoding *ApILP* and *ApFoxO* were cloned and identified, and their contributions to the trehalose catabolism were assessed in *A. pernyi* pupae during diapause termination. Injection of bovine insulin and RNA interference (RNAi) experiments targeting the *ApILP* gene showed that *ApILP* promotes trehalose hydrolysis by upregulating both *ApTre-1A* and *ApTre-2*; moreover, bovine insulin injection also increases the activity of soluble and membrane-bound trehalase during diapause termination. In addition, the results of overexpressing and silencing *ApFoxO* suggest a functional association between *ApFoxO* and the trehalose level in the haemolymph. The present study contributes to a better understanding of the roles of *ApILP* and *ApFoxO* in regulating the trehalose metabolism during diapause termination in the pupae of *A. pernyi*; it lays a foundation for later research on the diapause termination mechanism of *A. pernyi*, and facilitates improved utilisation of *A. pernyi* resources.

## 2. Materials and Methods

### 2.1. Insects

The larvae and pupae of the bivoltine strain *Qing No. 6* of *A. pernyi* were obtained from the Sericultural Research Institute of Liaoning Province (Fengcheng, China). Eight fifth-instar larvae aged 10 days were employed and various tissues were dissected, including fat bodies, hemocytes, silk glands, midgut, Malpighian tubules, and epidermis. The fourth- and fifth-instar larvae, early pupae, 5-day-old and 10-day-old pupae, and 1-day-old adults were also collected. Dissected tissues were immediately frozen in liquid nitrogen and then stored at −80 °C until use. Diapausing pupae were placed under a short-day photoperiod of L:D 9:15 at 25 °C for 2 weeks to ensure that they remained in the state of diapause until their use in experiments. To break diapause, pupae were exposed to a long-day photoperiod of L:D 17:7 at 25 °C. The diapause state was determined based on the transparency of the epicranium [17,24].

### 2.2. Total RNA Extraction and Cloning of Full-Length cDNA

Total RNA was extracted from the fat bodies, haemocytes, silk glands, midgut, Malpighian tubules, and epidermis of fifth-instar larvae after these tissues were ground in liquid nitrogen using the RNAiso Plus reagent (Takara, Shiga, Japan), in accordance with the manufacturer’s instructions. First-strand cDNA was synthesized using the PrimeScript™ 1st strand cDNA Synthesis Kit (Takara, Shiga, Japan), in accordance with the manufacturer’s instructions. To clone the *ApILP* gene, the primers *ApILP*-F and *ApILP*-R were used for amplification. The PCR reaction contained 0.3 μL of TaKaRa Ex Taq, 5 μL of 10× Ex Taq buffer, 4 μL of dNTP mixture, 33.7 μL of dH_2_O, 2 μL of cDNA, and 2.5 μL of each primer (10 μM). The reaction procedure was as follows: 40 cycles of denaturation at 94 °C for 30 s, annealing at 45 °C for 30 s, and elongation at 72 °C for 1 min. To clone the *ApFoxO* gene, PCR was performed with the primers *ApFoxO*-F and *ApFoxO*-R. Amplification cycling parameters were 40 cycles at 95 °C for 30 s, 58 °C for 30 s, and 72 °C for 2 min, which was followed by an extension at 72 °C for 10 min. The amplification products were analysed on 1% agarose gels stained with ethidium bromide. The amplification products were then purified using a DNA gel extraction kit (TransGen, Beijing, China), and were subsequently sequenced by Sangon Biotech (Sangon Biotech, Shanghai, China).

### 2.3. Sequence Alignment and Phylogenetic Tree Construction

For homologous alignment, the deduced amino acid sequence, encoded by *ApILP* or *ApFoxO*, was compared to those of other insect species, which were downloaded from the GenBank database. Amino acid sequence alignments were performed using Clustal X software (version 2.0). Analyses of the molecular mass (kDa) and isoelectric point (pI) of proteins were carried out using the ExPASy online server (https://www.expasy.org/; accessed on 17 October 2020). The signal peptide was predicted using the SignalP 5.0 server (http://www.cbs.dtu.dk/services/SignalP/; accessed on 17 October 2020). The phylogenetic tree was constructed in MEGA (version 7) using the neighbour-joining method with 1000 bootstrap replicates.

### 2.4. Administration of Bovine Insulin

Bovine insulin (Sigma-Aldrich, St. Louis, MO, USA) was first dissolved in 1 M HCl; then, the pH was adjusted to 3 using 0.5 M NaOH; finally, the solution was diluted to the desired concentration using 100 mM PBS buffer (pH 7.4). Various amounts of bovine insulin (0, 1, 5, 10, 20, and 40 μg/10 μL) were injected into diapausing *A. pernyi* pupae using a Hamilton microsyringe. Equal volumes of PBS buffer were used as controls. All treated pupae were incubated at 25 °C and L:D 9:15, and adult emergence was recorded in each group. The experiment was conducted three times, and each group contained 20 individuals.

As treatment with a dose of 10 μg bovine insulin could prompt subsequent development in nearly all diapausing *A. pernyi* pupae (as mentioned above), 10 μg (10 μL) of bovine insulin was injected, and its effect on the trehalose catabolism was assessed. Equal volumes of PBS buffer were used as controls. Treated *A. pernyi* pupae were collected, and their gene expression, trehalose and glucose levels, and trehalase activity were analysed at selected time points up to 18 d post-injection.

### 2.5. RNA Interference Targeting ApILP, ApFoxO, and ApTres

With the gene-specific primers ds*ApILP*-F and ds*ApILP*-R (Table S1), containing the T7 polymerase promoter sequence, a 327 bp dsRNA fragment that specifically targets *ApILP* (ds*ApILP*) was synthesised. The Promega T7 expression kit (Promega, Madison, WI, USA) was used for in vitro transcription, following the manufacturer’s protocol. The final concentration of ds*ApILP* was adjusted to 5 µg/µL with nuclease-free water. To perform RNAi, pupae were first placed under the long-day photoperiod conditions for 17 d to break the diapause; then, 50 µg of ds*ApILP* was injected into the abdomens of pupae to silence *ApILP*. Equal amounts of dsRNA targeting the enhanced green fluorescent protein gene (ds*EGFP*), derived from a 435 bp fragment of the *EGFP* gene, were injected into pupae in the control group. In this experiment, each group contained eight individuals, and was prepared in triplicate. The weight of the *A. pernyi* pupae was ~8 g. All treated pupae were collected for further analysis at 24, 36, 48, and 72 h after dsRNA injection.

The 435 bp dsRNA fragment targeting *ApFoxO* was prepared using ds*ApFoxO*-F and ds*ApFoxO*-R (Table S1), following the same method used for the synthesis of ds*ApILP*. Similar to the RNAi experiment that targeted *ApILP*, 50 µg (10 µL) of ds*ApFoxO* was injected into the abdomens of diapausing pupae, and equal amounts of ds*EGFP* were injected (~8 g mean fresh body weight) into pupae in the control group. In this experiment, each group also contained eight individuals, and was prepared in triplicate. At 48 h post-injection, all treated pupae were collected for further analysis.

To generate specific dsRNA fragments targeting *ApTres*, the lengths of the homologous dsRNAs for *ApTre-1A*, *ApTre-1B*, and *ApTre-2* used in this study were 762, 779, and 773 bp, respectively. Three pairs of primers (Table S1) with the T7 polymerase promoter sequence were designed according to these regions. The dsRNA fragments targeting ds*ApTres* were synthesized in vitro using the Promega T7 expression kit. For RNA interference, pupae were first placed under the long-day photoperiod conditions for 17 d to break diapause; then, 50 µg (10 µL) each of ds*ApTre-1A*, ds*ApTre-1B*, and ds*ApTre-2* were injected into the abdomens of the pupae. Equal amounts of ds*EGFP* were injected (approximately 8 g mean fresh body weight) into pupae in the control group. In this experiment, each group also contained eight individuals, and was prepared in triplicate. At 48 h post-injection, all treated pupae were collected for further analysis.

### 2.6. Baculovirus-Mediated Overexpression of ApFoxO in A. pernyi Pupae In Vivo

The *A. pernyi* nucleopolyhedrovirus (ApNPV) expression vector system has been previously used for the stable expression of foreign genes in *A. pernyi* pupae [25,26]. To transiently overexpress *ApFoxO* in vivo, the gene was cloned into the ApNPV transfer vector pApM748BE, and 1 μg of pApM748BE/*ApFoxO* was co-transfected with 0.5 μg of the genomic DNA of ApNPV-Δ*ph*/*egfp*^+^ into Tn-Hi5 cells (1 × 10^6^) using Cellfectin II. After incubation at 27 °C for 5 d, the supernatant of the cell culture was harvested, and then 100 µL of the supernatant was injected into *A. pernyi* pupae to generate the recombinant virus ApNPV-Δ*ph*/Δ*egfp/ApFoxO*^+^. Modified plaque purification was used to isolate the recombinant virus. Selected cells containing the recombinant virus were picked up using sterile Pasteur pipettes, resuspended in 100 µL of TNM-FH serum-free medium, and then the viral supernatant was injected into *A. pernyi* pupae. After incubation at 25 °C for 7 d, the tissue (including haemolymph and fat bodies of the infected pupae) was used to extract viral DNA using GenElute™ Mammalian Genomic DNA Miniprep Kits (Sigma-Aldrich, St. Louis, MO, USA). The purity of the recombinant virus was assessed by PCR with Pap-seqF and Pap-seqR primers. The titre of the recombinant virus in the haemolymph of infected pupae was estimated according to the method of Reed and Muench [27]. The supernatant of the haemolymph of infected pupae was diluted 10 times with TNM-FH serum-free medium, filter-sterilized through a 0.22 μm filter (Millipore), and then injected into pupae (10^5^ pfu/pupa), which had been subjected to the long-day photoperiod for 14 days. Infected pupae were incubated at 25 °C and were collected for further bioassays 6 d after injection of the recombinant virus ApNPV-Δ*ph*/Δ*egfp/ApFoxO*^+^. Pupae injected with ApNPV-Δ*ph*/*egfp*^+^ served as controls.

### 2.7. Quantitative Real-Time PCR

Quantitative real-time PCR (qRT-PCR) was conducted using a LightCycler^®^ 96 (Roche, Basel, Switzerland) with SYBR^®^ Premix Ex Taq™ II (Takara, Shiga, Japan). The following conditions were applied: pre-incubation at 95 °C for 30 s, followed by 40 cycles of denaturation at 95 °C for 5 s, and annealing and elongation at 60 °C for 30 s. All gene-specific primers used for qRT-PCR are listed in Table S1. The relative expression levels of target genes were normalised with the two stable reference genes *actin* and *RP49*, and expression levels were calculated using the 2^−ΔΔct^ method [28].

### 2.8. Measurement of Trehalose and Glucose Levels

The haemolymph of *A. pernyi* pupae was collected, and trehalose and glucose were extracted. The extraction was performed following a previously described method [17,29]. Extracted samples were analysed with an Agilent HPLC system equipped with an evaporative light-scattering detector and a sugar-D, NH_2_-MS packed column (4.6 mm I.D. × 250 mm, COSMOSIL, Nacalai Tesque, Kyoto, Japan). The mobile phase consisted of a mixture of acetonitrile and water (80:20 *v*/*v*), and the flow rate was 1 mL/min. The quantities of trehalose and glucose present in the samples were calculated according to standard solutions of trehalose and glucose, respectively.

### 2.9. Measurement of Trehalase Activity

The activity of soluble and membrane-bound trehalase was determined according to a previously described procedure [11,30]. Briefly, after removal of the puparium, the whole bodies of *A. pernyi* pupae were ground in 20 mM PB buffer, sonicated for 30 s, and centrifuged at 4 °C and 12,000× *g* for 30 min. The supernatant was directly used to measure the activity of soluble trehalase, and the precipitate was used for a membrane-bound trehalase activity assay after resuspension in 20 mM PB buffer; then, 200 µL of soluble or membrane-bound trehalase fraction extract was mixed with 250 µL of 40 mM trehalose and 550 µL of 20 mM PB buffer. The reaction mixture was incubated at 25 °C for 30 min, and then in boiling water for 5 min. After centrifugation, the supernatant was used to measure the glucose content with a Glucose Assay Kit (Comin, Suzhou, China).

### 2.10. Statistical Analysis

Three biological and three technical replicates were used for each experiment. All data are presented as means ± standard errors, determined using SPSS software version 22.0 (SPSS Inc., Chicago, IL, USA). Differences between groups were determined using Student’s *t*-tests (for comparisons between two mean values) or by one-way analysis of variance (ANOVA) followed by a least significant difference (LSD) test (for comparisons between multiple mean values). Differences at *p* < 0.05 were considered significant.

## 3. Results

### 3.1. Cloning and Expression Profiles of ApILP and ApFoxO

Based on our previous transcriptome data on *A*. *pernyi* (unpublished), the full-length cDNAs of *ApILP* and *ApFoxO* were obtained. The *ApILP* sequence consists of 327 bp and encodes a polypeptide of 108 amino acids, showing homology (20–38%) to other insect ILPs (Figures S1A and S2A). The open reading frame of *ApFoxO* consists of 1563 bp, and was translated into a protein of 520 amino acids. BLAST analysis showed that the deduced amino acid sequence of *ApFoxO* has high homology (81–89%) with other insects’ FoxOs (Figures S1B and S2B).

**Figure 1:**
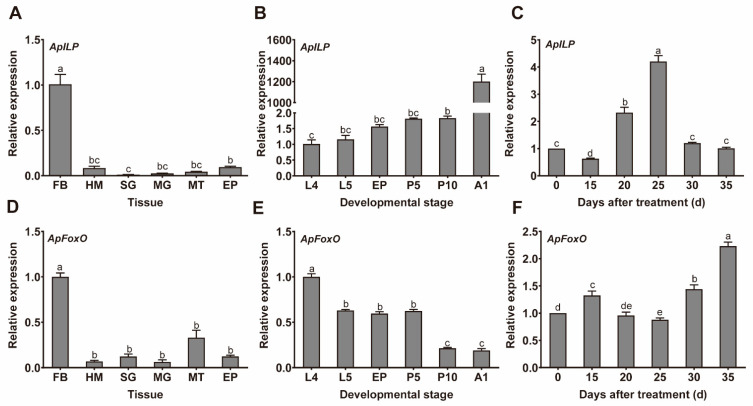
Temporal and spatial expression patterns of *ApILP* and *ApFoxO* in *Antheraea* *pernyi*. (**A**,**D**) Relative expression levels of *ApILP* and *ApFoxO*, respectively, in different tissues of fifth-instar larvae; abbreviations: fat body (FB); hemocyte (HM); silk gland (SG); midgut (MG); Malpighian tubules (MT); epidermis (EP). (**B**,**E**) Expression profiles of *ApILP* and *ApFoxO*, respectively, at different developmental stages; abbreviations: 4th- and 5th-instar larvae (L4 and L5, respectively); early pupae (EP); 5-day-old and 10-day old pupae (P5 and P10, respectively); 1-day-old adults (A1). (**C**,**F**) Changes in the relative expression levels of *ApILP* and *ApFoxO*, respectively, after diapausing pupae were subjected to long-day photoperiod (L:D 17:7) treatment to break diapause. Based on the transparency of the epicranium, treated pupae were collected for qRT-PCR analysis on indicated days (15, 20, 25, 30, and 35 d) after long-day photoperiod (L:D 17:7) treatment. Diapausing pupae (day 0) were used as a control group. The period of the long-day photoperiod treatment incorporated two phases: the diapause termination phase (which continued until day 20 of treatment), and the post-termination phase (which started on day 25 and ended on day 35 of treatment). The mRNA levels were normalised to the reference genes *actin* and *RP49*. Data represent the means of three independent experiments, and error bars are SEs. Different letters above the error bars indicate significant differences at *p* < 0.05.

The expression patterns of both genes were analysed from different tissues of fifth-instar larvae and at their different developmental stages. The tissue expression data show that *ApILP* and *ApFoxO* expression were detected in all tested tissues; however, the expression levels of *ApILP* and *ApFoxO* were significantly higher in the fat body than in other tissues (Figure 1A,D). The transcripts of *ApILP* and *ApFoxO* were also expressed at all developmental stages of *A. pernyi*. *ApILP* expression was significantly higher in the adult stage compared with other stages, while *ApFoxO* exhibited significantly higher expression levels at the larval stage (Figure 1B,E). In addition, *ApILP* expression increased significantly on days 20 and 25 after long-day photoperiod treatment to break diapause. However, the expression of *ApFoxO* followed an up–down–up pattern during the long-day photoperiod treatment period, suggesting that *ApILP* and *ApFoxO* might be involved in the regulation of the developmental process at diapause termination and post-termination phases (Figure 1C,F).

### 3.2. Bovine Insulin Induces Diapause Termination

**Figure 2:**
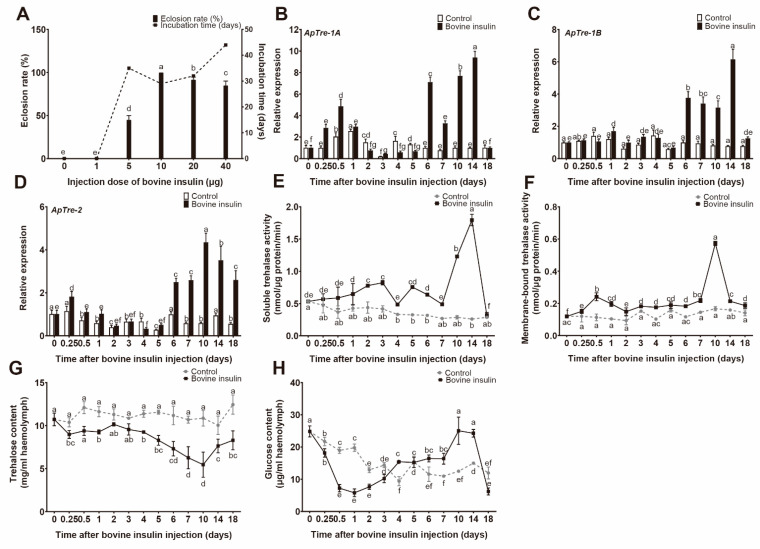
Bovine insulin injection induced diapause termination and enhanced trehalose catabolism in *A. pernyi* diapausing pupae. (**A**) Effect of different doses of bovine insulin on breaking the diapause of *A. pernyi* pupae. Diapausing pupae were exposed to a short-day photoperiod of L:D 9:15 at 25 °C after bovine insulin injection. Eclosion rates were recorded. Each group consisted of 20 individuals, and error bars indicate the mean ± SE of three replicates. (**B**–**D**) Relative expression levels of *ApTre-1A*, *ApTre-1B*, and *ApTre-2*, respectively, induced by bovine insulin injection. The mRNA levels were normalised to the reference genes *actin* and *RP49*, and pupae treated with PBS buffer served as controls (CK). The mRNA levels of *ApTres* in pupae before treatment (0 h) were defined as 1. (**E**,**F**) Changes in soluble and membrane-bound trehalase activity, respectively, caused by bovine insulin injection. (**G**,**H**) Influence of bovine insulin treatment on haemolymph trehalose and glucose levels, respectively. The dotted lines represent the control group (which received PBS buffer), while the solid lines represent the bovine-insulin-treated group. Data are means of three independent experiments, and error bars are SEs. Different letters above the error bars indicate significant differences at *p* < 0.05.

The insect IIS pathway is involved in the regulation of insect development, longevity, cell cycle, metabolism, and female reproduction [31]. Previous studies have reported that the repression of ILP is associated with the generation of a diapause phenotype, suggesting that ILP plays an important role in the regulation of diapause in insects [32,33,34]. Because bovine insulin has 21.24% amino acid sequence homology with *ApILP*, the effect of bovine insulin injection on breaking diapause was investigated (Figure 2A). At a bovine insulin injection dose of 1 μg, treatment failed to produce any clear result. With increasing dose, the eclosion rate was significantly stimulated, and the best emergence rate (100%) was achieved at 10 μg. However, when the applied dose exceeded 10 μg, the emergence rate started to decline slightly (95% at 20 μg and 85% at 40 μg). Incubation times varied from 44 ± 4 days (40 μg) to 29 ± 2 days (10 μg). These results show that bovine insulin injection can effectively promote diapause termination in *A. pernyi* pupae.

### 3.3. Bovine Insulin Enhances Trehalose Catabolism

Given the effect bovine insulin has on the breaking of diapause in *A. pernyi*, the next test assessed whether trehalose catabolism was affected by bovine insulin. qRT-PCR analysis showed that bovine insulin injection triggered significant increases in *ApTre-1A*, *ApTre-1B*, and *ApTre-2* mRNA levels. Compared with the control group, injection of bovine insulin induced a rapid increase of *ApTre-1A* expression 6–24 h after injection; however, *ApTre-1A* expression decreased 2–5 d after treatment, and again dramatically increased from 6 to 14 d after bovine insulin injection, peaking at 14 d post-injection (where the level was 9.40-fold higher than that of the control group) (Figure 2B). Similarly, the first increase in *ApTre-2* expression was observed 6–24 h after injection of bovine insulin; then, *ApTre-2* expression was significantly upregulated from 6 to 18 d after bovine insulin treatment, and the mRNA level of *ApTre-2* was 7.34-fold higher than that of the control group at 10 d post-injection (Figure 2D). In addition, the relative expression of *ApTre-1B* significantly increased on days 1, 6, 7, 10, 14, and 18 after bovine insulin injection, in comparison with the control group (Figure 2C). Injection of bovine insulin significantly elevated the enzyme activity of soluble and membrane-bound trehalase in diapausing *A. pernyi* pupae, compared with pupae from the control group (which received PBS buffer only). Soluble trehalase activity gradually increased with bovine insulin injection from 6 h to 14 d post-injection, especially from 7 d to 14 d, during which time a sharp increase was observed; however, the magnitude of the increase diminished clearly at 4 d and 7 d (Figure 2E). Similarly, the activity of membrane-bound trehalase increased after bovine insulin injection, especially at 12 h and at 10 d after treatment (Figure 2F).

In addition, injection of bovine insulin caused a significant decrease in trehalose concentration in diapausing *A. pernyi* pupae. The trehalose concentration of the control group remained relatively constant at 11.16 ± 0.67 mg/mL, while it decreased from 10.72 mg/mL (0 h) to 5.46 mg/mL (10 d) following bovine insulin treatment (Figure 2G). Moreover, the glucose content of pupae in the control group decreased slightly and fluctuated over a small range during the study period; however, the concentration of glucose in pupae treated with bovine insulin followed a down–up–down pattern, which is roughly the opposite trend compared with the trehalose content (Figure 2H).

### 3.4. ApILP RNAi Suppresses the Utilization of Trehalose

**Figure 3:**
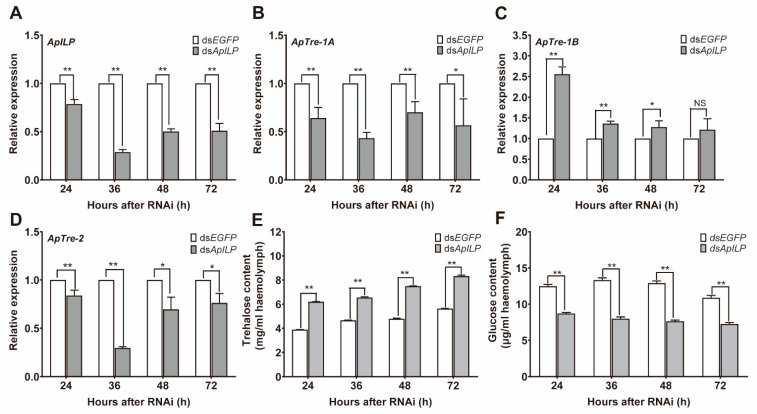
Effects of *ApILP*-specific RNA interference (RNAi) on trehalose metabolism in diapausing *A. pernyi* pupae during diapause termination. To effectively break diapause, diapausing *A. pernyi* pupae were placed under the long-day photoperiod conditions (L:D 17:7) for 17 days before receiving either ds*ApILP* or ds*EGFP* treatments. (**A**–**D**) Relative expression levels of *ApILP*, *ApTre-1A*, *ApTre-1B*, and *ApTre-2*, respectively. (**E**,**F**) Changes in trehalose and glucose levels, respectively, in the haemolymph. mRNA levels were normalised to those of the reference genes *actin* and *RP49*, and the group injected with ds*EGFP* served as controls. Data are means of three independent experiments, and error bars are SEs. Single asterisks (*p* < 0.05) and double asterisks (*p* < 0.01) represent significant differences between the ds*EGFP* and ds*ApILP* groups in the same time period.

Bovine insulin injection induces the breakdown of trehalose, suggesting that *ApILP*—a homologue of vertebrate insulin—might be involved in the modulation of haemolymph trehalose levels in *A. pernyi*. The *ApILP* gene was silenced by an RNAi experiment to further confirm its role in the regulation of the trehalose catabolism. After injection of *ApILP*-specific dsRNA, expression levels of *ApILP* were 21.4%, 71.2%, 50.0%, and 49.1% lower than those of the control group at 24 h, 36 h, 48 h, and 72 h, respectively. This suggests that *dsApILP* injection significantly suppressed the mRNA level of *ApILP*, and that the suppression lasted for at least 72 h, leading to an effective RNAi response (Figure 3A). After injection of *ApILP*-specific dsRNA, the transcription levels of *ApTre-1A* and *ApTre-2* decreased (Figure 3B,D), while the transcription level of *ApTre-1B* increased (Figure 3C). Under this RNAi condition, the trehalose levels in the haemolymph exceeded those of the control group (Figure 3E). In contrast, ds*ApILP* treatment resulted in a significant reduction in the glucose content compared with the control group (Figure 3F). These results indicate that the suppression of *ApILP* expression inhibits trehalose utilisation.

### 3.5. Effect of Overexpressing and Silencing ApFoxO on Trehalose Metabolism

**Figure 4:**
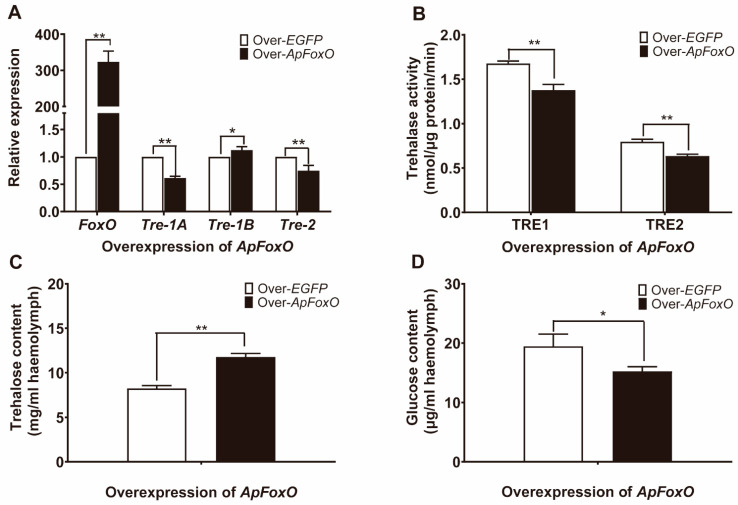
Overexpression of *ApFoxO* suppressed trehalose catabolism in *A. pernyi* pupae during diapause termination. All diapausing pupae were placed under the long-day photoperiod conditions (L:D 17:7) for 14 days to break diapause; then, they were injected with the recombinant virus ApNPV, containing either *ApFoxO* or *EGFP* cDNA under the promoter of the viral polyhedrin (polh) gene. Six days after the injection of baculovirus, pupae were collected for further analysis. (**A**) Relative expression levels of *ApFoxO*, *ApTre-1A*, *ApTre-1B*, and *ApTre-2*. (**B**) Changes in soluble (TRE1) and membrane-bound (TRE2) trehalase activity. (**C**,**D**) Changes in trehalose and glucose levels, respectively, in the haemolymph. mRNA levels were normalised to those of the reference genes *actin* and *RP49*, and the group used for the overexpression of *EGFP* served as controls. Data are means of three independent experiments, and error bars are SEs. Single asterisks (*p* < 0.05) and double asterisks (*p* < 0.01) represent significant differences between the ds*EGFP* and ds*ApILP* groups in the same time period.

**Figure 5:**
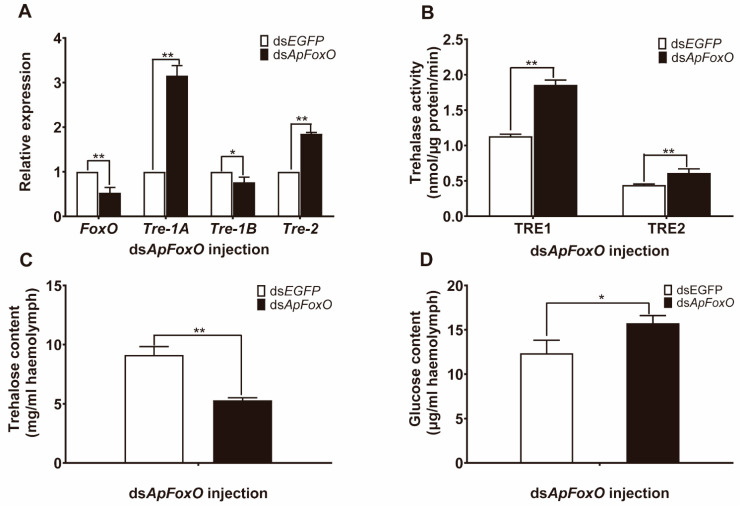
Effects of *ApFoxO*-specific RNAi on trehalose metabolism in diapausing *A. pernyi* pupae. (**A**) Relative expression levels of *ApFoxO*, *ApTre-1A*, *ApTre-1B*, and *ApTre-2* at 48 h after ds*ApFoxO* injection in comparison to controls (ds*EGFP* injection). (**B**) Changes in soluble (TRE1) and membrane-bound (TRE2) trehalase activity caused by ds*ApFoxO* or ds*EGFP* treatment at 48 h post-injection. (**C**,**D**) Changes in trehalose and glucose content, respectively, in the haemolymph at 48 h post-injection. mRNA levels were normalised to those of the reference genes *actin* and *RP49*, and the group injected with ds*EGFP* served as controls. Data are means of three independent experiments, and error bars are SEs. Single asterisks (*p* < 0.05) and double asterisks (*p* < 0.01) represent significant differences between the ds*EGFP* and ds*ApFoxO* groups in the same time period.

To further confirm that the IIS pathway is involved in the regulation of trehalose levels, the gene *ApFoxO*—the downstream target of insulin signalling—was overexpressed and silenced. Compared with the injection of *EGFP*-baculovirus, injection of *ApFoxO*-baculovirus into *A. pernyi* pupae led to a dramatic increase in the *ApFoxO* mRNA levels, and downregulated the mRNA levels of *ApTre-1A* and *ApTre-2*. However, *ApTre-1B* expression was slightly upregulated after overexpression of *ApFoxO* (Figure 4A). Furthermore, a decrease in both soluble and membrane-bound trehalase activity was observed (Figure 4B), which resulted in an increased trehalose level and a decreased glucose level, when the gene of *ApFoxO* was overexpressed (Figure 4C,D). In contrast, knockdown of *ApFoxO* in diapausing pupae induced upregulation of *ApTre-1A* and *ApTre-2* expression, but slightly downregulated *ApTre-1B* expression compared with the control group (Figure 5A). In addition, increases in soluble and membrane-bound trehalase activity accelerated the conversion of trehalose into glucose when *ApFoxO* expression was silenced (Figure 5B–D). These results suggest that *ApFoxO* expression is positively correlated with trehalose levels by decreasing the mRNA levels of *ApTre-1A* and *ApTre-2* and their corresponding activities.

### 3.6. Knockdown of ApTres Suppresses Trehalose Catabolism

**Figure 6:**
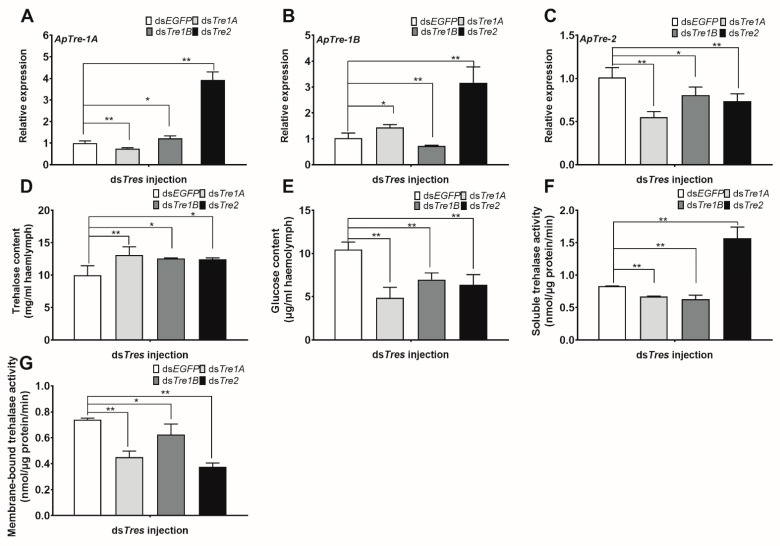
Effects of RNAi targeting the three *ApTres* on trehalose catabolism in *A. pernyi* pupae. (**A**–**C**) Relative expression levels of *ApTre-1A*, *ApTre-1B*, and *ApTre-2*, respectively, at 48 h after RNAi targeting the three *ApTres*. (**D**,**E**) Changes in trehalose and glucose content, respectively, in the haemolymph at 48 h after RNAi targeting the three *ApTres*. (**F**,**G**) Changes in soluble and membrane-bound trehalase activity, respectively, at 48 h after RNAi targeting the three *ApTres*. mRNA levels were normalised to those of the reference genes *actin* and *RP49*, and the group injected with ds*EGFP* served as controls. Data are means of three independent experiments, and error bars are SEs. Single asterisks (*p* < 0.05) and double asterisks (*p* < 0.01) represent significant differences between the ds*EGFP* and ds*ApTres* groups in the same time period.

To investigate the function of these *ApTres* genes and their relationship in the regulation of trehalose levels in the haemolymph, pupae were injected with specific dsRNAs targeting *ApTre-1A*, *ApTre-1B*, and *ApTre-2*. Pupae were collected for further analysis at 48 h after injection. The pupae injected with *EGFP*-specific dsRNA served as controls. Compared with the control group, the expression of *ApTre-1A*, *ApTre-1B*, and *ApTre-2* decreased to 74.5%, 72.3%, and 73.7%, respectively, at 48 h after the injection of the corresponding dsRNA. This indicates that RNAi targeting each *ApTre* was successful in each group (Figure 6A–C). In addition to injection of the corresponding dsRNA, injection of *ApTre-1A* and *ApTre-1B* dsRNAs also decreased *ApTre-2* expression (Figure 6C). The expression of *ApTre-1A* and *ApTre-1B* increased significantly after *ApTre-2* RNAi injection. In addition, *ApTre-1A* showed a slight increase after *ApTre-1B* RNAi injection, while *ApTre-1B* also increased after *ApTre-1A* RNAi injection (Figure 6A,B). These changes in the expression levels of *ApTres* indicate possible inter-regulations between different *ApTres*. Silencing *ApTre-1A* and *ApTre-1B* decreased the activity of both soluble and membrane-bound trehalase; however, silencing *ApTre-2* decreased membrane-bound trehalase activity but increased soluble trehalase activity. These changes in trehalase activity were generally consistent with their corresponding expression level trends (Figure 6F,G). Moreover, knockdown of *ApTre-1A*, *ApTre-1B*, or *ApTre-2* resulted in an obvious increase in trehalose levels in the haemolymph, but caused a reduction in haemolymph glucose levels, compared with ds*EGFP* treatment (Figure 6D,E).

## 4. Discussion

ILP is involved in the regulation of the metabolism, growth, reproduction, and lifespan of insects [35]. Many insect ILPs have been identified in different species, including the fruit fly *D. melanogaster* [6], the silkworm *B. mori* [36], the cotton leafworm *Spodoptera littoralis* [37], the beet armyworm *Spodoptera exigua* [38], the brown planthopper *Nilaparvata lugens* [39], and the mosquito *Anopheles gambiae* [40], as well as the locusts *Locusta migratoria* [41], and *Schistocerca gregaria* [42]. Insect ILPs share a similar structure with vertebrate insulin, and its precursors comprise a signal peptide, B chain, interconnecting C peptide, and A chain. Except for insulin growth factors, the ILP precursors are generally processed into an active form that is A- and B-chain-linked by disulphide bonds after removal of the C peptide [43]. In the present study, an ILP of *A. pernyi* was cloned and identified based on its high amino acid sequence similarities with other known insect ILPs. In addition, the identified ILP contained a signal peptide and a B–C–A chain, which includes conserved cysteine residues and dibasic cleavage sites for the proteolytic cleavage of C peptide. This finding suggests that *ApILP* may be activated by the removal of the C peptide and the formation of disulphide bridges of the B–A chain (Figure S2A).

Many insects have been reported to possess multiple ILPs. These ILPs are at least in part expressed in a tissue-specific or stage-specific manner, which is associated with their distinct functions [43]. A specific ILP is predominantly expressed in the fat body [42,44]. In *B. mori*, the IGF-like peptide Bommo-IGFLP (*BIGFLP*) is mainly produced in the fat body during pupa–adult development, and its secretion is stimulated by the ecdysteroid 20-hydroxyecdysone (20E) [45]. Similarly, *Drosophila* insulin-like peptide 6 (*DILP6*) is expressed in the fat body during the postfeeding stage in response to ecdysteroids [46]. In this study, *ApILP* was also predominantly expressed in the fat body at the pupal stage (Figure S3A). Moreover, the mRNA level of *ApILP* in the fat body increased ~3.26-fold after diapausing pupae were treated with exogenous 20E (Figure S3B); this suggests that *ApILP* is regulated by 20E at a transcriptional level, which coincides with the high expression level of *ApILP* during the late larval stage. Thus, it can be assumed that *ApILP* is a counterpart of this specific ILP, akin to *BIGFLP* and *DILP6*, and is functionally important to the *A. pernyi* fat body. The insect fat body, equivalent to vertebrate liver and adipose tissue, is a major site of nutrient storage and energy metabolism. Existing literature has shown that the fat body plays a central role in the integration of hormonal and nutritional signals, regulating insect development and behaviour, including pupal diapause [47]. In diapausing *H. armigera* pupae, the fat body can exert an effect on the brain’s regulatory centre by altering the levels of sugar and tricarboxylic acid (TCA) metabolites [48]. This evidence suggests that the fat body might play an important role in diapause termination. Furthermore, this study showed that an increase in *ApILP* expression is associated with diapause termination in diapausing *A. pernyi* pupae (Figure 1C). Thus, it can be assumed that the fat-body-derived ILP of *A. pernyi* might play a potential role in diapause termination.

It has been reported that treatment with vertebrate insulin can lead to diapause termination via activation of the prothoracic gland in diapausing *P. brassicae* and *Antheraea mylitta* pupae [23,49]. Alternatively, exogenous insulin directly exerts its effects on target tissues and improves their metabolic rate, which also stimulates diapause termination [50]. In the present study, injection of bovine insulin could effectively trigger the adult emergence of diapausing *A. pernyi* pupae (Figure 2A). Diapause termination is related to the energy metabolism of diapausing individuals, and is characterized by an elevated respiration rate [8]. As the only enzyme that hydrolyses trehalose into glucose, trehalase regulates both energy metabolism and glucose generation via trehalose catabolism [51]; this suggests that trehalase might play an important role in diapause termination and subsequent development. The results of the present study show that changes in gene expression differ slightly between *ApTre-1A*, *ApTre-1B*, and *ApTre-2* following bovine insulin injection; however, the overall expression trends of these genes increased, which may be related to the functional specificity of trehalase (Figure 2B–D). Exogenous insulin induced a significant increase in soluble and membrane-bound trehalase activity in diapausing *A. pernyi* pupae, thereby leading to a significant decline in trehalose levels in the haemolymph (Figure 2E–G). Following bovine insulin treatment, the levels of glucose in the haemolymph showed a trend of first decreasing and then increasing, which was followed by a decrease in comparison to the PBS-buffer-treated group (Figure 2H); this trend was likely caused by subsequent changes in glucose levels in pupae treated with bovine insulin. The consumption of glucose was accelerated with the increased metabolic respiration of diapausing pupae, while the elevated trehalase activity promoted the hydrolysis of trehalose into glucose. This resulted in an increased glucose concentration, and a decrease in trehalase activity led to a decline in glucose levels in the haemolymph. These results indicate that vertebrate insulin elevates trehalose catabolism in diapausing *A. pernyi* pupae. In agreement with these results, injection of vertebrate insulin suppressed haemolymph trehalose levels in lepidopteran insects, including *S. exigua* and *M. vitrata* [20,38]. Thus, the obtained results suggest that vertebrate insulin could enhance trehalose catabolism and promote pupa-to-adult development in diapausing *A. pernyi* pupae. We previously studied the action of exogenous 20E on trehalose catabolism in diapausing *A. pernyi* pupae [17]. Similarities exist between the effects of exogenous 20E and bovine insulin, according to their contribution to the elevated trehalose catabolism. Injections of 20E and bovine insulin both induced the upregulation of *ApTre-1A* and *ApTre2* which, in turn, increased the enzyme activity of soluble and membrane-bound trehalase, leading to a decline in haemolymph trehalose levels. Similar to the effects of bovine insulin and 20E on trehalose in diapausing pupae, the regulation of trehalose by juvenile hormone analogues in diapausing adults has also been reported. For example, methoprene treatment could effectively disrupt imaginal diapause and caused a marked increase in locomotory activity accompanied by a decline in trehalose activity in females of the apple blossom weevil *Anthonomus pomorum* [52]. This evidence indicates that the enhanced trehalose catabolism might play an important role in the diapause termination process.

Because vertebrate insulin can modulate trehalose levels, the effect of *ApILP* on trehalose catabolism was investigated via an RNAi experiment. The RNAi of *ApILP* decreased the mRNA levels of *ApTre-1A* and *ApTre-2*, which led to an increase in the trehalose content and, in turn, decreased the glucose levels (Figure 3). These results are consistent with a previous study that reported that the knockdown of an ILP gene (*SeILP1*), which is mainly expressed in the fat body, induced a significant increase in circulating trehalose levels in *S. exigua* [38]. It has been reported that, in *D. melanogaster*, trehalase exists in two forms, including cytoplasmic and putative secreted forms, and that a compensation mechanism exists between trehalase isoforms [53]. In the present study, *ApTre-1B* expression increased significantly after ds*ApILP* injection, which might be a form of compensation for the decreases in *ApTre-1A* and *ApTre-2* expression, providing the energy needed for survival (Figure 3C). It has been reported that, in *A. pernyi*, trehalose concentration remains at a high level during diapause maintenance, but declines when diapause is broken [16,17]. This change in trehalose concentration indicates that the reduction in trehalose concentration is associated with diapause termination in *A.* *pernyi*. Based on the contribution of *ApILP* to regulating trehalose levels, and the trend of *ApILP* expression during diapause termination, we speculate that *ApILP* is responsible for the downregulation of trehalose levels in the haemolymph of *A. pernyi* by increasing the expression of *ApTre-1A* and *ApTre-2* during diapause termination.

Insulin signalling is considered a diapause regulator, as it controls developmental processes and glycolipid metabolism in insects [5,54,55]. A high insulin level represses FoxO activity through p-Akt and, subsequently, activates many target genes that promote insect development. In contrast, shutdown of insulin signalling represses PI3K/Akt which, in turn, increases the activity of FoxO, and leads to the generation of the diapause phenotype [56]. Accumulation of FoxO was reported in the brain of diapausing *Helicoverpa armigera* pupae, and overexpression of FoxO impedes larval development and extends the lifespan at the early larval phase [57]. Similarly, in diapausing *Laodelphax striatellus* nymphs, FoxO mRNA expression and protein levels were found to be much higher than those of non-diapausing nymphs [58]; these results corroborate the involvement of FoxO in diapause regulation. The present study showed that overexpression of *ApFoxO* decreased the mRNA levels of *ApTre-1A* and *ApTre-2*, and suppressed the activity of soluble and membrane-bound trehalase during diapause termination; this led to an increase in trehalose levels and a decrease in glucose levels (Figure 4). In contrast, silencing *ApFoxO* increased *ApTre-1A* and *ApTre-2* expression, as well as the activity of soluble and membrane-bound trehalase, which accelerated the hydrolysis of trehalose in diapausing pupae (Figure 5). Consistent with the results of the present study, knockdown of *LSFoxO* significantly increased trehalase activity and shortened the diapause duration in *L. striatellus* nymphs [58]. Additionally, knockdowns of *ApTre-1A* and *ApTre-2* both induced an increase in *ApTre-1B* expression (Figure 6B), suggesting that changes in *ApTre-1B* expression might be affected by the expression of *ApTre-1A* and *ApTre-2* after overexpressing and silencing *ApFoxO* (Figure 4A and Figure 5A). In summary, *ApFoxO* may modulate trehalose catabolism, thus affecting pupa–adult development in *A. pernyi*.

## 5. Conclusions

**Figure 7:**
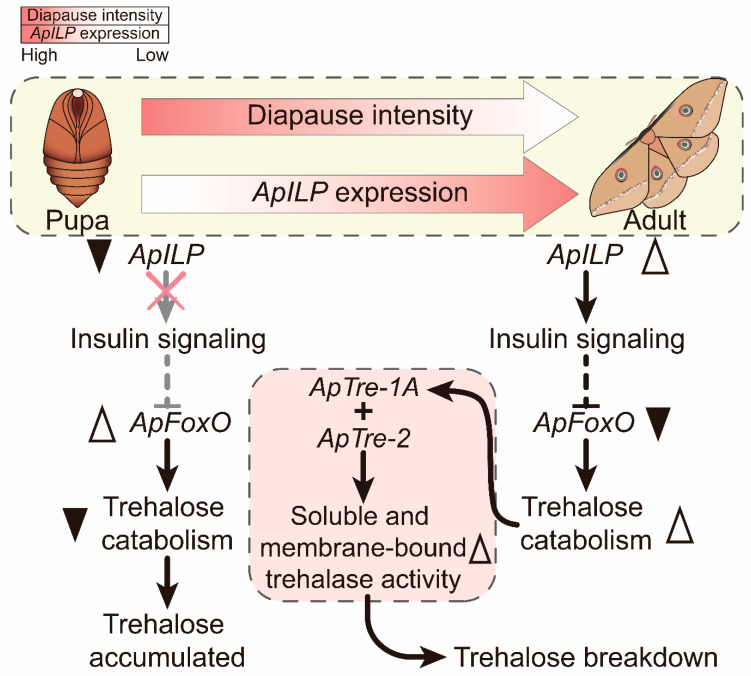
Schematic depiction showing that *ApILP* and *ApFoxO* are involved in the regulation of the trehalose catabolism in the diapause termination process in *A. pernyi* pupae. When *A. pernyi* pupae undergo diapause, the expression of *ApILP* is relatively low, and insulin signalling is repressed which, in turn, stimulates an increase in *ApFoxO* activity; then, *ApFoxO* reduces trehalase activity, thus suppressing trehalose hydrolysis. However, the expression of *ApILP* increases during diapause termination, which inhibits *ApFoxO* activity through the insulin/insulin-like growth factor signalling pathway. Subsequently, the increase in *ApTre1A* and *ApTre2* expression triggers the activity of soluble and membrane-bound trehalase, which enhances trehalose catabolism and accelerates trehalose breakdown. Black indicates gene activation, while grey indicates deactivation.

In conclusion, the present study shows that an increase in *ApILP* expression is associated with diapause termination in *A. pernyi* pupae. The application of the *ApILP* homologous vertebrate insulin effectively stimulated diapause termination and elevated the trehalose catabolism of diapausing *A. pernyi* pupae. In contrast, the RNAi of *ApILP* suppressed trehalose hydrolysis through the downregulation of *ApTre-1A* and *ApTre-2* expression and the reduction in the activity of soluble and membrane-bound trehalase during diapause termination. In addition, overexpression of the transcription factor *ApFoxO* induced trehalose accumulation during diapause termination, while the silencing of *ApFoxO* enhanced trehalose breakdown in diapausing pupae. As the main transcriptional effector of the IIS pathway, *ApFoxO* exhibited a contrasting effect on the trehalose catabolism to that of *ApILP* (Figure 7).

## Data Availability

The data presented in this study can be found in the supplementary materials.

## Supplementary Materials

The following are available online at https://www.mdpi.com/article/10.3390/insects12090784/s1: Figure S1: Phylogenetic analysis of *ApILP* and *ApFoxO*; Figure S2: Alignment analysis of *ApILP* and *ApFoxO*; Figure S3: Expression of *Antheraea pernyi ApILP*; Table S1: Primer sequences used in this study.
