# Supplementary material for: Insulin-Like Peptide and FoxO Mediate the Trehalose Catabolism Enhancement during the Diapause Termination Period in the Chinese Oak Silkworm (Antheraea pernyi)
Source: Insects. 2021 Sep 1;12(9):784. doi: 10.3390/insects12090784 (PMC8472214; doi:10.3390/insects12090784)
Supplement: Supplementary file 1 [file insects-12-00784-s001.zip › insects-1329986-supplementary.pdf]

Figure S1

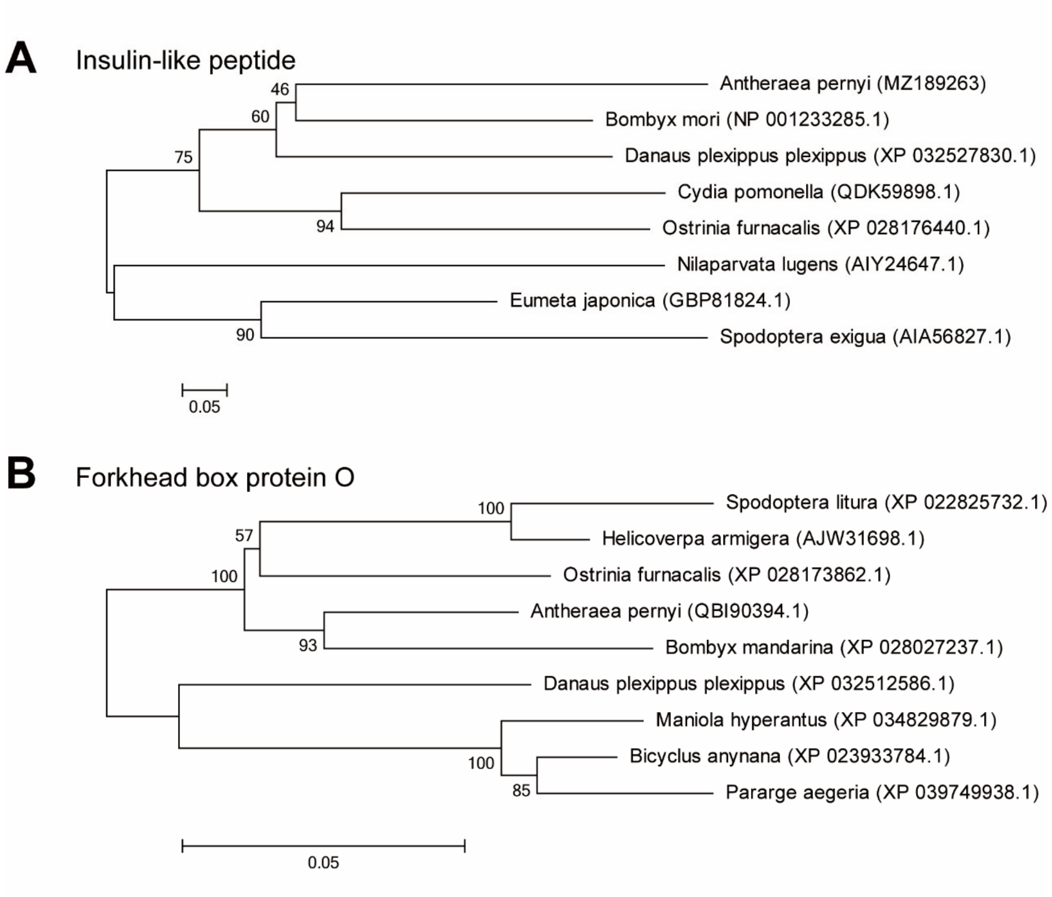

Figure S1. Phylogenetic analysis of *ApILP* and *ApFoxO*. Phylogenetic trees of *ILP* (A) and *FoxO* (B) in *Antheraea pernyi* and other insects. Trees were constructed with the neighbour-joining method based on amino acid sequences. The accession numbers of the sequences used for the analysis are shown in parentheses. Boot strap values for 1000 trials are indicated at each node.

Figure S2

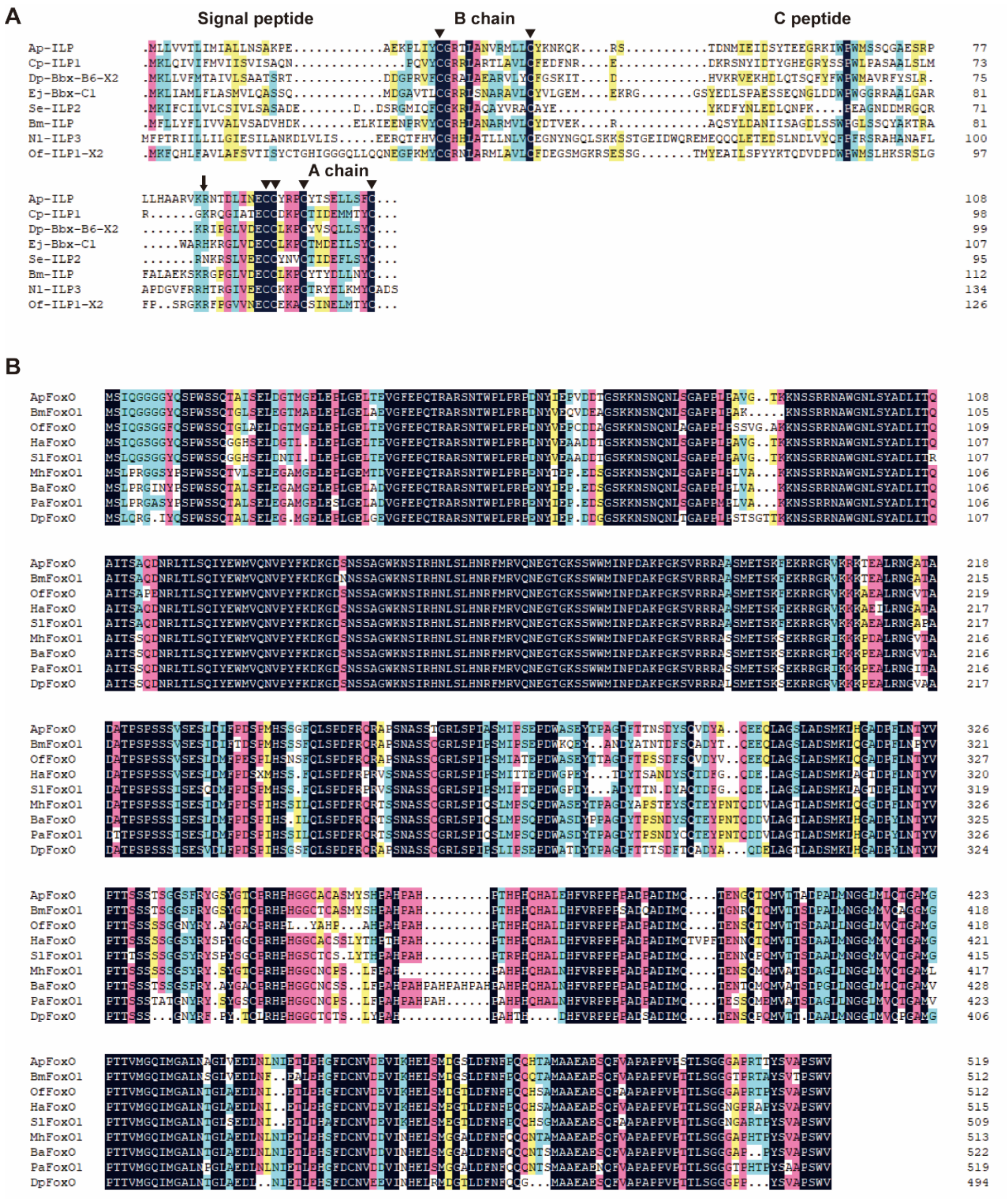

Figure S2. Alignment analysis of *ApILP* and *ApFoxO*. Multiple alignment of *ApILP* (A) and *ApFoxO* (B) amino acid sequences with their homologues. Identical amino acid residues are highlighted with the same colour. Conserved cysteine residues at the B and A chain of *ApILP* are indicated with a triangle (▼). The putative cleavage site of *ApILP* is indicated with an arrowhead (↓). The following amino acid sequences were used in the alignment: *Bombyx mori* (Bm-ILP, NP_001233285.1), *Cydia pomonella* (CpILP, QDK59898.1), *Danaus plexippus plexippus* (Dp-Bbx-B6-X2, XP_032527830.1), *Eumeta japonica* (Ej-Bbx-C1, GBP81824.1), *Ostrinia furnacalis* (Of-ILP1-X2, XP_028176440.1), *Spodoptera exigua* (Se-ILP2, AIA56827.1), *Nilaparvata lugens* (Nl-ILP3, AIY24647.1), *Bicyclus anynana* (BaFoxO, XP_023933784.1), *Bombyx mandarina* (BmFoxO1, XP_028027237.1), *Danaus plexippus plexippus* (DpFoxO, XP_032512586.1),

Helicoverpa armigera (HaFoxO, AJW31698.1), *Maniola hyperantus* (MhFoxO1, XP_034829879.1), *Ostrinia furnacalis* (OfFoxO, XP_028173862.1), *Pararge aegeria* (PaFoxO1, XP_039749938.1), and *Spodoptera litura* (SlFoxO1, XP_022825732.1).

Figure S3

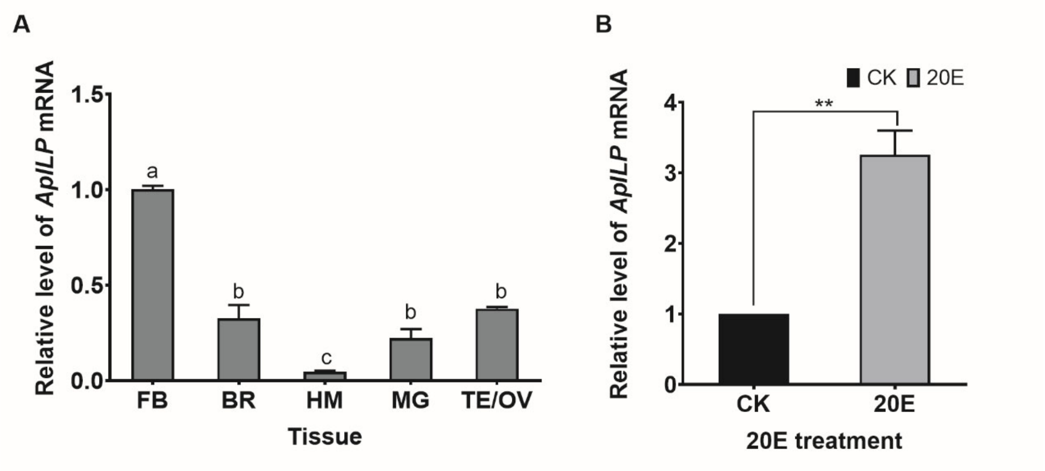

Figure S3. Expression of *Antheraea pernyi ApILP*. (A) Tissue distribution of *ApILP* in 10-day-old pupae. Abbreviations: Fat body (FB), brain (BR), hemocyte (HM), midgut (MG), testes and ovaries (TE/OV). (B) Changes in the relative expression levels of *ApILP* in the fat body on day 1 after diapausing pupae were injected with 20E (20 μg).

Table S1

Table S1. Primer sequences used in this study

Primer name	Nucleotide sequences (5′–3′)
PCR	
ApILP-F	AAATATGCGGCCGCAAATGTTGCTGGTCGTGAC
ApILp-R	CCCAAGCTTTTAACAAAAGGAGAGTAATTCG
ApFoxO-F	CCATATGATGTCTATACAAGGAGGTGGCGGTTAC
ApFoxO-R	GCGGCCGCGTGGACCCAGGAGGGGGC
pApM748BE-EGFP-F	CTCGGATCCATGGTGAGCAAGGGCGAGG
pApM748BE-EGFP-R	CTCGAATTCTTACTTGTACAGCTCGTCC
pApM748BE-FoxO-F	GGATCCATGTCTATACAAGGAGGTGGCGGT
pApM748BE-FoxO-R	TCTAGATCAATGATGATGATGATGATGGTGGACCCAGGAGGGGGC
Pap-seq-F	TTAACGCTTAGCCAGCAGCAG
Pap-seq-R	TTCTTTACCGCTCCAGTTGAC
qRT-PCR	
Ap-actin-qF	ACCACACCTTCTACAATGAGC
Ap-actin-qR	ACGTCTCGAACATGATCTGTG
ApRP49-qF	AAGACCCGTCACATGCTACC
ApRP49-qR	GCGTTCGACGATTAACTTCC
ApTre-1A-qF	ACGAAGACTACACCAATGCTC
ApTre-1A-qR	ACCAACGAGATGAAAAGTCCC
ApTre-1B-qF	AGACGGAGTCTGGTACGATTA
ApTre-1B-qR	TAACGCGGAGCATCGTATTC
ApTre-2-qF	TGAAGGGTCCGAGTTTGAAG
ApTre-2-qR	CTGACGCCCACTGATGAAA
ApILP-qF	ATGGATGTCGTCTCAAGGC
ApILp-qR	CTAGTGTAGCAAGGTCTGTAGC
ApFoxO-qF	CAGATTCACCAATGCACAGC
ApFoxO-qR	TCTGGTTCTGAAGGAATCATAGAC
EGFP-qF	AGTTGTACTCCAGCTTGTGC
EGFP-qR	GACGGCAACTACAAGACCC
RNAi	
dsEGFP-F	TAATACGACTCACTATAGGGAGACCACCTACGGCAAGCTGACCCTGAAGT
dsEGFP-R	TAATACGACTCACTATAGGGAGAGCCGTCGCCGATGGGGGTGTTCTGCTG
dsApILP-F	TAATACGACTCACTATAGGATGTTGCTGGTCGTGACG
dsApILP-R	TAATACGACTCACTATAGGTTAACAAAAGGAGAGTAAT
dsApFoxO-F	TAATACGACTCACTATAGGGAGAGACGGAAATCCGGGGACAAC
dsApFoxO-R	TAATACGACTCACTATAGGGAGACAAGCCATCACCTCCGCTCA
dsApTre1A-F	TAATACGACTCACTATAGGGTACCAGGAACGTAGCCAACC
dsApTre1A-R	TAATACGACTCACTATAGGGAAGTAATCAAGGACGCGTGG
dsApTre1B-F	TAATACGACTCACTATAGGGTGGGGATACGTACCTAACGG
dsApTre1B-R	TAATACGACTCACTATAGGGGTAACGCGGAGCATCGTATT
dsApTre2-F	TAATACGACTCACTATAGGGTCGTTTCCGCGAGTTCTATT
dsApTre2-R	TAATACGACTCACTATAGGGACATCCTCGTGCCAGAAAAC

Ap, Antheraea pernyi; actin, actin gene GU073316; RP49, ribosomal protein 49 gene DQ296005; ILP, insulin-like peptide gene MZ189263; FoxO, forkhead box protein O gene MZ572537; Tre-1A, soluble trehalase 1A gene KU977455; Tre-1B, soluble trehalase 1B gene KU977456; Tre-2, membrane-bound trehalase gene KU977457; EGFP, enhanced green fluorescent protein gene 20473140. F indicates forward, and R indicates reverse.
